# Too Insecure to Be a Leader: The Role of Attachment in Leadership Emergence

**DOI:** 10.3389/fpsyg.2020.571401

**Published:** 2020-11-17

**Authors:** Yang Yang, Yongli Wang, Hailing Lu, Ling Tan

**Affiliations:** ^1^Business School, Sun Yat-sen University, Guangzhou, China; ^2^School of Economics and Management, Nanjing University of Science and Technology, Nanjing, China

**Keywords:** attachment, leadership emergence, negative emotion, initiating structure, socioanalytic theory

## Abstract

The antecedents of leadership emergence have received increasing attention over the past decades. Extant work has found that traits that involve *getting along* with other members in social relations can help employees emerge as leaders. However, attachment has been ignored, even though it can provide a distinct relational perspective to *getting along*. This study investigates the relationship between attachment and leadership emergence as well as the mediating role of negative emotion and the moderating role of initiating structure in the relationship. Specifically, based on multisource data of 100 employees and their supervisors, the results reveal that avoidant attachment and anxious attachment exert a negative impact on leadership emergence via negative emotion. Moreover, the mediating effect on the above relationship is weaker when employees are at a high initiating structure level. The findings imply that insecurely attached employees can also be leaders if they expend more effort and focus more on task completion.

## Introduction

Leadership emergence refers to “the degree to which a person who is not in a formal position of authority influences the other members of a group” (Côté et al., 2010, p. 496). With more autonomy and decision-making responsibility provided for employees (Guzzo and Dickson, 1996), leadership emergence has a greater impact on employee performance and team effectiveness. Specifically, at the individual level, employees high in informal leadership will perform better as a result of team members’ support and high work motivation. At the team level, leadership emergence can also promote team cooperation and improve team performance (Taggar et al., 1999; Cogliser et al., 2012; Zhang et al., 2012; Liu et al., 2018). Given the importance of informal leadership, a growing body of literature has explored the antecedents of leadership emergence in recent years (Ensari et al., 2011; Tuncdogan et al., 2017).

Over the last 30 years, increasing evidence has shown that traits can explain some of the variance in leadership emergence (Zaccaro et al., 1991). In other words, leadership emergence depends on other members’ perception of whether the employee holds the prototypical traits of a leader (Lord and Maher, 1990). Existing research has revealed that traits involving *getting along* with other members increase opportunities for occupational success. Hence, *getting along* is a critical explanation of emergence as a leader (Hogan, 1996; Taggar et al., 1999; Hogan and Holland, 2003). Many studies have explored the influence of the Big Five, leadership motives, and narcissism on leadership emergence, which involve broad traits with multifaceted natures. Only some facets of these variables reflect *getting along* at work (Luria and Berson, 2013; Marinova et al., 2013; Hu et al., 2019). Despite the importance of these variables, exploring traits that are “more directly related to how people relate to other people,” such as attachment, can add to our understanding of the nature of *getting along* (Richards and Schat, 2011, p169).

Attachment is defined as the propensity to seek and develop emotional bonds with others (Bowlby, 1969), which are more directly related to how people relate to each other than broad traits (Richards and Schat, 2011). Noftle and Shaver (2006) found that attachment can show more significant predictive power for a relationship than the Big Five, while many argue that attachment can endow a unique significance to *getting along* (Yip et al., 2018). However, although attachment may be an important determinant of leadership emergence, it has not yet received due attention. Only a few studies have investigated how attachment exerts an impact on leadership emergence (Mikulincer and Florian, 1995; Berson et al., 2006), and the issue of why it does so remains untilled. Only by exploring how, why, and when attachment exerts an impact on leadership emergence can we adopt effective means to promote employees’ leadership.

Attachment has two dimensions: avoidant attachment and anxious attachment. Employees with high avoidant attachment or high anxious attachment are considered to display insecure attachment. For an adequate understanding of attachment, it is necessary to explore the mediating mechanism. Attachment has effects on emotional response patterns (Collins, 1996). Specifically, avoidant and anxious attachment can lead to more negative emotion (Richards and Schat, 2011). According to socioanalytic theory, negative emotion is not aligned with *getting along* (Staw et al., 1994). Hence, employees will not be prone to emerge as leaders when their negative emotion is increased by insecure attachment.

We argue that insecurely attached employees are seen as leaders significantly less often than securely attached employees due to negative emotion. Can insecurely attached employees never be leaders? According to socioanalytic theory, *getting along* with other members and *getting ahead* among other members can both contribute to leadership emergence (Hogan and Holland, 2003; Marinova et al., 2013; Hu et al., 2019). Employees may be simultaneously low in *getting along* and high in *getting ahead*, and a high level of *getting ahead* may buffer the negative impact of a low level of *getting along* on leadership emergence. Initiating structure reflects the degree to which an employee is oriented toward goal attainment (Fleishman, 1973), which corresponds to *getting ahead* (Hogan and Holland, 2003). We can assume that, although insecure attachment and negative emotions are not conducive to *getting along*, attention to task completion and goal attainment can facilitate *getting ahead* and buffer the negative impact. In other words, initiating structure will weaken the relationship among insecure attachment, negative emotion, and leadership emergence.

**FIGURE 1:**
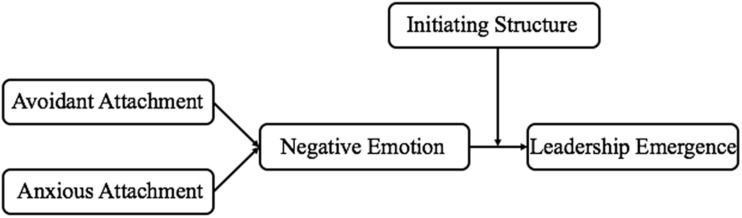
Conceptual model of the study.

Our study investigates how, why, and when insecure attachment impairs leadership emergence (Figure 1). This study contributes to the existing literature in several ways. First, attachment can more directly reflect how people get along with other people than the Big Five (Richards and Schat, 2011). This study examine how *getting along* exerts an impact on leadership emergence through a distinct relational perspective. Second, we use socioanalytic theory to examine the underlying mechanism of negative emotion between attachment and leadership emergence. The findings could deepen our understanding of the processes by which attachment influences leadership emergence. Moreover, by investigating the moderating effect of initiating structure, we find a complementary effect of *getting along* and *getting ahead*. The findings imply that insecurely attached employees can also be leaders if they expend more effort and focus more on task completion.

## Theory and Hypotheses

### Attachment and Negative Emotion

Attachment, the special emotional connection between caregivers and infants, has an effect across different relationships over the lifespan of an individual (Bowlby, 1969). According to the internal working model, attachment, which consists of avoidant attachment and anxious attachment, can impact beliefs about the self and others throughout a person’s lifespan (Collins, 1996). Specifically, avoidantly attached individuals believe others are untrustworthy or malevolent, whereas anxiously attached individuals believe that they are essentially unlovable (Brennan et al., 1998; Mikulincer and Shaver, 2015). In sum, attachment can activate individuals’ views of themselves and others, which in turn exert a direct impact on cognitive response and emotional response (Collins, 1996; Collins et al., 2004).

According to the internal working model, avoidantly and anxiously attached individuals experience greater negative emotion than individuals who are securely attached (Mikulincer and Shaver, 2007). Attachment has both direct and indirect effects on emotional response. First, attachment is affect-laden and can directly trigger emotional responses (Collins, 1996). Insecurely attached individuals appraise others as untrustworthy and unavailable when dealing with interpersonal relationships (Frazier et al., 2015). Hence, the negative emotion of insecurely attached individuals may be automatically activated when bonding with others (Collins, 1996). Second, attachment can influence negative emotion indirectly by cognitive response, one aspect of which is explanatory style. Individuals with different explanatory styles are predisposed to different emotions. Insecurely attached individuals hold negative beliefs about themselves and others; thus, they are prone to explain events in a negative way (Collins, 1996). Insecurely attached individuals’ pessimistic explanations will result in negative emotion. Therefore, we can argue that avoidant and anxious individuals are characterized by a range of negative emotions in the workplace (Magai et al., 1995).

The relationship between insecure attachment and negative emotion has been well studied. Insecure attachment is associated with more frequent and intense negative emotions (Feeney, 1999; Richards and Schat, 2011). Specifically, avoidant attachment is associated with greater disgust, shame, anxiety, disgust, contempt, fear, hostility, and envy (Magai and McFadden, 1995; Consedine and Magai, 2003; Paech et al., 2016), whereas anxiously attached individuals experience greater fear, shame, hostility, envy, depression, and anger (Mikulincer et al., 1990; Magai and McFadden, 1995; Paech et al., 2016). In sum, we assume that avoidantly and anxiously attached individuals have more negative emotions than securely attached individuals.

Hypothesis 1: Avoidant and anxious attachments are positively associated with employees’ negative emotions.

### Attachment, Negative Emotion, and Leadership Emergence

Leadership emergence depends on other members’ perceptions of whether an employee holds the prototypical traits of a leader (Lord and Maher, 1990; Epitropaki and Martin, 2004). The utilization of socioanalytic theory for leadership emergence can provide specific insights into leadership emergence on the basis of traits and can demonstrate the perspectives of both actors and observers (Hogan and Holland, 2003; Marinova et al., 2013). According to socioanalytic theory, all employees work in groups. The social processes of *getting along* with other team members and *getting ahead* among other team members can jointly influence career success (Hogan and Holland, 2003). To get along, employees should be friendly and positive. They must “demonstrate interpersonal skill, work with others, show positive attitudes, and share credit.” To get ahead, employees should be responsible and have initiative. They must “work with energy, exhibit effort, value productivity, and show concern for quality” (Hogan and Holland, 2003, p. 105). *Getting along* and *getting ahead* can both help employees obtain higher status in groups (Hogan, 1982) and contribute to leadership emergence.

Employees with more negative emotions cannot be positive and friendly, which is not conducive to *getting along* with others. Specifically, employees with negative emotion show less favorable altruism and cooperation with other members; correspondingly, employees with negative emotion receive more unfavorable feedback from other members than coworkers with positive emotion (Staw et al., 1994). Hence, the expression of negative emotion leads to unfavorable interaction with team members and is detrimental to *getting along* with other members.

Employees’ negative emotions are not conducive to *getting along*, which in turn destroys leadership emergence. Past studies have found that emotion plays a critical role in leadership (Gooty et al., 2010). Employees with negative emotion are less preferred as leaders and are judged to be less effective in their leadership roles than positive individuals (Schaumberg and Flynn, 2012).

Hypothesis 2: Negative emotion is negatively associated with leadership emergence.

Employees with avoidant and anxious attachments experience more negative emotions, which influences their ability to get along with other members. *Getting along* is an important determinant of leadership emergence (Hogan and Holland, 2003; Marinova et al., 2013; Hu et al., 2019). In summary, we assume that attachment influences leadership emergence via negative emotion.

Hypothesis 3: Negative emotion mediates the relationship between avoidant/anxious attachment and leadership emergence.

### The Moderating Role of Initiating Structure

According to socioanalytic theory, employees can be perceived as leaders by both *getting along* and *getting ahead* (Hogan and Holland, 2003; Marinova et al., 2013; Hu et al., 2019). *Getting along* focuses on building and maintaining interpersonal relationships, whereas *getting ahead* focuses on task completion and goal attainment. *Getting along* and *getting ahead* jointly influence leadership emergence by unique mechanisms (Marinova et al., 2013). Extant work has indicated that *getting along* and *getting ahead* may be incompatible (Hogan and Holland, 2003). This means that employees may be simultaneously low in *getting along* and high in *getting ahead*. A high level of *getting ahead* may buffer the negative impact of a low level of *getting along* on leadership emergence. Hence, the negative relationship between negative emotion and leadership emergence may be weaker when an employee emphasizes *getting ahead*.

Initiating structure is defined as the degree to which an employee is oriented toward goal attainment (Fleishman, 1973; Mikulincer et al., 1990), which corresponds to the effort to *get ahead* in the workplace (Hogan and Holland, 2003). When employees have a relatively low initiating structure, *getting along* becomes the only path to emerge as a leader. Thus, negative emotion may exert a more powerful impact on leadership emergence. When employees have a relatively high initiating structure, they may be at a high level of *getting ahead*; thus, *getting along*, and the expression of negative emotion, may play a less important role in determining the emergence of leadership. Hence, we assume that initiating structure can weaken the effect of negative emotion on leadership emergence. The leadership emergence of employees with a relatively low initiating structure is more likely to be affected by negative emotion than the leadership emergence of those with a high initiating structure.

Hypothesis 4: Initiating structure moderates the relationship between negative emotion and leadership emergence such that the negative relationship is weaker among followers with high rather than low levels of initiating structure.
Hypothesis 5: Initiating structure moderates the strength of the mediated relationships between avoidant/anxious attachment and leadership emergence via negative emotion such that the mediated relationship will be weaker under a high initiating structure than under a low initiating structure.

## Materials and Methods

### Sample and Procedure

We invited part-time MBA graduate students from five classes to participate in the study. Part-time MBA students take weekend classes at the university and work on weekdays. They all have more than 3 years of work experience and formal supervisors. One hundred pairs of MBA students (female, 51%; male, 49%) and their immediate supervisors agreed to take part in the study.

Each student received a packet containing a pair of matching follower and leader questionnaires. The MBA followers completed the follower questionnaire, and then, they asked their supervisors to fill out the leader questionnaire. Each supervisor received a short letter explaining the study and assuring them of the confidentiality of responses as well as an envelope with a unique seal.

### Measures

#### Attachment

Attachment was evaluated with the Experience in Close Relationships (ECR) scale (Brennan et al., 1998). The ECR scale contains 36 items to identify avoidant and anxious attachments. The avoidant attachment items include “It helps to turn to my romantic partner in times of need.” The anxious attachment items include “I need many reassurances that I am loved by my partner.” Responses are based on a 7-point scale ranging from 1 (totally disagree) to 7 (totally agree). The coefficient alpha was 0.880 for avoidant attachment, and for anxious attachment, it was 0.872.

The ECR scale includes items that describe employees’ feelings in close relationships. According to the internal working model, attachment can reflect general beliefs about the self and others throughout a person’s lifespan (Collins, 1996), and these beliefs remain relatively stable across different relationships over the life course (Mikulincer and Shaver, 2003). Hence, the ECR scale specific to close relationships can reflect employees’ attitudes and behavior patterns in work settings. The ECR scale is a valid and most widely used measurement of attachment (Mikulincer and Shaver, 2007; Yip et al., 2018). Many studies have investigated the impact of attachment on work settings using the ECR scale (Dahling and Librizzi, 2015; Reizer, 2019). Hence, it is theoretically reasonable to employ the ECR scale to assess employees’ attachment style in work settings.

#### Negative Emotion

We used the positive and negative affect schedule (PANAS) scale to measure employees’ negative emotion. Existing research has found that the PANAS scale is highly internally consistent, largely uncorrelated, and stable at appropriate levels over a 2-month time period (Watson et al., 1988). The negative emotion dimension consists of 10 emotional adjectives, such as “nervous” and “distress.” Employees rate themselves on a 7-point frequency ranging from 1 (never) to 7 (always). The coefficient alpha for the current study was 0.902.

#### Initiating Structure

Employee initiating structures were measured by a leader behavior description questionnaire (Stogdill, 1963). The 10-item initiating structure dimension includes “I let group members know what is expected of them.” Employees rate themselves on a 7-point scale ranging from 1 (totally disagree) to 7 (totally agree). The coefficient alpha for the current study was 0.912.

#### Leadership Emergence

To rate employees’ leadership emergence, the supervisors completed the 3-item scale, including “potential for advancement in your organization” (Marinova et al., 2013). Immediate leaders rated the items on a 7-point scale ranging from 1 (totally disagree) to 7 (totally agree). The coefficient alpha for the current study was 0.883.

Leadership emergence has typically been measured in larger groups (Judge et al., 2002). The measures were designed mainly for leaderless workgroups or short-lived groups. However, extant research has revealed that informal leadership can also emerge and exert an impact on others in a well-established organizational context despite the formal presence of supervisors (Wheelan and Johnston, 1996). In this circumstance, “supervisors are most likely to be knowledgeable about leadership emergence processes” (Marinova et al., 2013, p. 1263). We conducted this study on employees with existing supervisors. Thus, it is rational that supervisors rated the potential of the employee become an effective leader and study leadership emergence in pairs.

#### Control Variables

We controlled for the sex (0 = *male*, 1 = *female*), age (1 = *20–29 years old*, 2 = *30–39 years old*, 3 = *40–49 years old*, 4 = *50* + *years old*) and education (1 = *bachelor’s degree*, 2 = *college degree*, 3 = *master’s degree*) of the participants in our analyses. In addition, on the basis of socioanalytic theory, consideration reflected the effort to get along (Hogan and Holland, 2003), which might exert an impact on leadership emergence. Thus, we measured consideration with a leader behavior description questionnaire (Stogdill, 1963). The consideration dimension contains 10 items. Sample items include “I do little things to make it pleasant to be a member of the group” and “I treat all group members as my equals.” The coefficient alpha in this study was 0.890.

## Results

### Confirmatory Factor Analysis

We conducted confirmatory factor analyses at an individual level using Mplus 7.4 to test the measurement model. We specified avoidant attachment, anxious attachment, negative emotion, initiating structure, and leadership emergence as separate factors. Compared with the small sample size in this study, there were too many parameters. Hence, we could not assess a complete item-level confirmatory factor analysis (CFA). We used item parcels instead (Little et al., 2002; Moore et al., 2019). We randomly parceled off avoidant attachment, anxious attachment, negative emotion, and initiating structure into three indicators.

The fit indices showed that the hypothesized five-factor model [χ*^2^* = 104.648, *df* = 80; root mean square error of approximation (*RMSEA*) = 0.056; comparative fit index (*CFI*) = 0.968; Tucker–Lewis index (*TLI*) = 0.958] yielded a better fit to the data than a four-factor model (avoidant attachment and anxious attachment) (χ*^2^* = 192.018, *df* = 84; *RMSEA* = 0.113; *CFI* = 0.860; *TLI* = 0.825) and a one-factor model (χ*^2^* = 551.719, *df* = 90; *RMSEA* = 0.226; *CFI* = 0.401; *TLI* = 0.302). These CFA results also provided support for the distinctiveness of the five study variables for subsequent analyses.

The means, standard deviations, and correlations among the variables in this study are presented in Table 1. Avoidant attachment (*r* = 0.321, *p* = 0.001) and anxious attachment (*r* = 0.298, *p* = 0.003) were positively related to negative emotion, and negative emotion was negatively related to leadership emergence (*r* = −0.327, *p* = 0.001).

**TABLE 1:** Means, standard deviations, and correlations between variables.

**Variable**	***M***	***SD***	**1**	**2**	**3**	**4**	**5**	**6**	**7**	**8**
1. Sex	0.490	0.502								
2. Age	1.640	0.560	−0.192							
3. Education	2.340	0.476	−0.112	0.085						
4. Consideration	5.303	0.732	−0.128	−0.054	−0.038					
5. Avoidant attachment	2.837	0.859	−0.139	0.025	0.137	−0.241*				
6. Anxious attachment	3.139	0.880	−0.059	−0.006	−0.054	−0.122	0.257**			
7. Negative emotion	2.803	1.005	−0.033	0.180	0.019	−0.038	0.321**	0.298**		
8. Initiating structure	4.979	0.896	−0.036	0.114	0.113	0.609**	−0.256*	−0.159	−0.161	
9. Leadership emergence	5.960	0.835	−0.009	0.048	0.068	0.270**	−0.283**	−0.201*	−0.327**	0.480**

*p < 0.05, **p < 0.01 (two-tailed).

### Hypothesis Tests

We used Mplus 7.4 to test the mediation and moderated mediation hypotheses (Table 2). First, we found that employees’ avoidant and anxious attachments were significantly and positively related to negative emotion (γ = 0.334, *SE* = 0.115, *p* = 0.004; γ = 0.272, *SE* = 0.110, *p* = 0.013). Therefore, hypothesis 1 was supported.

**TABLE 2:** Results of the structural equation model.

**Variables**	**Negative emotion**	**Leader emergence**
	**M1 γ (SE)**	**M2 γ (SE)**
Sex	0.132 (0.192)	−0.065 (0.155)
Age	0.345* (0.168)	0.052 (0.158)
Education	−0.028 (0.206)	0.101 (0.162)
Consideration	0.107 (0.157)	−0.011 (0.124)
Avoidant attachment	0.334** (0.115)	−0.126 (0.096)
Anxious attachment	0.272* (0.110)	−0.024 (0.088)
Negative emotion		−0.185* (0.083)
Initiating structure		0.363** (0.101)
Negative emotion × Initiating structure		0.135* (0.067)
R^2^	0.192	0.320

*p < 0.05, **p < 0.01 (two-tailed).

Second, the path coefficient for the effect of negative emotion on leadership emergence was significant (γ = −0.185, *SE* = 0.083, *p* = 0.026). The mediating effect of negative emotion was found in the relationship between avoidant attachment [indirect effect = 0.062, 95% CI = (−0.163, −0.010)]/anxious attachment [indirect effect = −0.050, 95% CI = (−0.143, −0.007)] and leadership emergence. Therefore, hypotheses 2 and 3 were supported.

**FIGURE 2:**
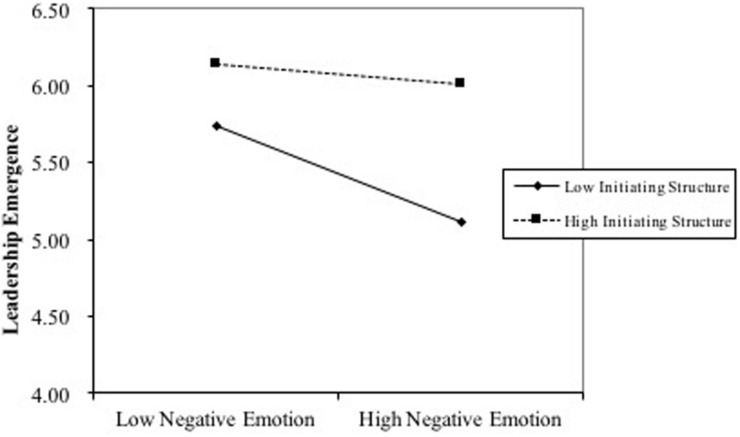
Moderating effect of initiating structure on the negative emotion–leadership emergence relationship.

Third, the results showed that initiating structure moderated the relationship between negative emotion and leadership emergence (γ = −0.135, *SE* = 0.067, *p* = 0.044) (Figure 2). When the initiating structure of employees was low, negative emotion had a significant negative effect on leadership emergence (γ = −0.306, SE = 0.107, *p* = 0.004). When the initiating structure of employees was high, negative emotion had no significant effect on leadership emergence (γ = −0.065, *SE* = 0.098, *p* = 0.511). Therefore, hypothesis 4 was supported.

Finally, the results supported the moderated mediation model. The mediation effect was relatively stronger when the initiating structure was low as opposed to when it was high. When the initiating structure of employees was low, avoidant and anxious attachments had an indirect effect on leadership emergence [indirect effect = −0.102, 95% CI = (−0.234, −0.027); indirect effect = −0.083, 95% CI = (−0.208, −0.015)]. When the initiating structure of employees was high, avoidant and anxious attachments did not have an indirect effect on leadership emergence [indirect effect = −0.022, 95% CI = (−0.109, 0.039); indirect effect = −0.018, 95% CI = (−0.093, 0.033)]. The difference between these indirect effects was significant [Δindirect effect = 0.081, 95% CI = (0.010, 0.204); Δindirect effect = 0.066, 95% CI = (0.005, 0.187)]. Therefore, hypothesis 5 was supported.

## Discussion

Drawing on the socioanalytic theory of leadership emergence, through a mediated moderation model, we examined the role of negative emotion and initiating structure in the relationship between attachment and leadership emergence. Our findings showed that avoidant and anxious attachments are detrimental to leadership emergence through negative emotion, and this relationship is moderated by initiating structure. Specifically, the mediated relationship is weaker under a high initiating structure than under a low initiating structure. These findings have both theoretical and practical implications.

### Theoretical Implications

The primary theoretical contribution of this study is that it identifies the effect of attachment on leadership emergence through a distinct relational perspective. Most prior research on leadership emergence has focused on broad traits. Compared with broad traits, attachment can more directly reflect how people relate to other people, which in turn influences work behavior (Richards and Schat, 2011). Attachment is critical to *getting along*; however, it has been ignored in the past. In this study, we examined how attachment influence leadership emergence. Moreover, due to the difference in avoidant and anxious attachments, we investigated the relationships respectively, this approach is superior to past research (Berson et al., 2006).

Second, on the basis of internal working model, attachment can activate individuals’ view of themselves and others and can have a direct impact on emotion (Collins, 1996). Past research has neglected the underlying mechanism between attachment and leadership emergence (Mikulincer and Florian, 1995; Berson et al., 2006). We explored the mediating process in our study and found that attachment exerted an impact on leadership emergence via negative emotion. These findings could deepen our understanding of the complexities of attachment for organizations and why attachment plays a role in the emergence of leadership.

Finally, according to socioanalytic theory, *getting along* and *getting ahead* can both contribute to leadership emergence (Hogan and Holland, 2003). In the past, researchers have distinguished the unique mechanisms behind leadership emergence (Marinova et al., 2013; Hu et al., 2019). However, the relationship between *getting along* and *getting ahead* has been neglected. Thus, we tested their complementary effect in our study. We found that *getting ahead* could make up for the lack of *getting along*; that is, the negative association between being inadequate at *getting along* and leadership emergence could be weakened by a high focus on *getting ahead*.

### Practical Implications

The findings of our study have several practical implications for both supervisors and employees. First, we found that employees with a high prevalence of negative emotion tend to be rated lower as emerging leaders. Negative emotion is detrimental to the career development of employees. This finding can inspire employees to try to control negative emotional expression in the workplace. Similarly, supervisors should realize that it is essential to establish efficient mechanisms to relieve negative emotion in the workplace.

The results also showed that insecurely attached employees are usually not regarded as leaders. However, most organizational studies have assumed that attachment is stable and consistent throughout an employee’s life span (Harms, 2011). Can insecurely attached employees never emerge as leaders? According to our study, *getting along* is not the only path to leadership emergence. Thus, it is important for insecurely attached employees to expend more effort on task completion and goal attainment. Our study can inspire employees who cannot get along with other members to pay more attention to *getting ahead*.

### Limitations and Future Research

Although our study used multisource data, it has several limitations. First, we used cross-sectional data in this study. Although traits preexist in emotional responses and behaviors according to the theoretical rationale, we cannot draw conclusions about causality without a longitudinal design. Reverse causation may exist between negative emotion and leadership emergence. Leaders’ assessments of their employees as “not having potential for advancement” may provide some clues to the target of the perception of negative evaluation, which leads to negative emotion. Future studies can design longitudinal research to rule out the possibility of reverse causation.

Second, the leadership emergence of employees was rated by their immediate supervisors in our study. Although “supervisors are most likely to be knowledgeable about leadership emergence processes in organization” (Marinova et al., 2013, p. 1263), leadership emergence focuses on the process of employees becoming influential with other members in a work team (Marinova et al., 2013). It may be preferable to assess group agreement on leadership emergence across multiple raters (Taggar et al., 1999). Future research can explore whether attachment exerts a similar impact on leadership emergence when it is measured by multiple members.

Third, we used the ECR scale to assess attachment, which contains items specific to close relationships. Although, according to the internal working model, attachment remains relatively stable across different relationships over the life course (Mikulincer and Shaver, 2003), the focus on close relationships may limit the ECR’s applicability to work settings. Future studies may consider the ECR-RS and state adult attachment measure (SAAM) scales to assess attachment style, which replace “close relationship” with “others” (Gillath et al., 2009; Fraley et al., 2011).

Fourth, the sample of this study was sourced from an MBA class, which included employees from different organizations. We omitted the effect of organizational culture. A study in the army found no difference between secure individuals and avoidant individuals in being nominated as a leader (Mikulincer and Florian, 1995), which indicates that the characteristics of leaders may be different in different organizational cultures. Future research could benefit by controlling the cultural variables in an organization.

Fifth, according to the internal working model, anxious and avoidant attachments involve unique traits with regard to emotion and cognition (Collins, 1996; Collins et al., 2004). Another limitation of this study is that we focused only on the emotional path of the relationship between attachment and leadership emergence. It would be fruitful for future studies to examine the mediating effect of cognition about the self and others. Avoidantly attached individuals perceive others as untrustworthy (Hazan and Shaver, 1987; Mikulincer, 1998; Mikulincer and Shaver, 2005), which may influence attribution and damage leadership emergence (Collins, 1996).

Finally, attachment is a dyadic attribute. Employees and supervisors possess attachment simultaneously. The attachment of both members of the dyad needs to be taken into account (Harms, 2011). For example, avoidant-attached leaders may prefer employees with an avoidant attachment style instead of a secure attachment style as leaders. Future studies can examine the effect of the attachment styles of a dyad on leadership emergence.

## Conclusion

Many previous studies on the antecedents of leadership emergence have focused on the Big Five. Attachment can reflect how people relate to other people and can provide a distinct relational perspective on leadership emergence. However, the perspective of the critical influence of attachment on leadership emergence is less prevalent. In this study, we examined the underlying mechanism of attachment and leadership emergence. We found that avoidant and anxious attachment can decrease leadership emergence via the expression of negative emotion. Furthermore, an initiating structure can weaken this relationship.

## Data Availability Statement

The raw data supporting the conclusions of this article will be made available by the authors, without undue reservation, to any qualified researcher.

## Ethics Statement

The study involving human participants was reviewed and approved by the Sun Yat-sen University Ethics Committee. The patients/participants provided their written informed consent to participate in this study.

## Author Contributions

YY was responsible for writing the initial draft of the manuscript. YW was responsible for the further modification and improvement of the manuscript. HL and LT provided critical revisions. All authors contributed to the article and approved the submitted version.

## Conflict of Interest

The authors declare that the research was conducted in the absence of any commercial or financial relationships that could be construed as a potential conflict of interest.
